# Regional Nanoindentation Properties in Different Locations on the Mouse Tibia From C57BL/6 and Balb/C Female Mice

**DOI:** 10.3389/fbioe.2020.00478

**Published:** 2020-05-15

**Authors:** Valentina Pepe, Sara Oliviero, Luca Cristofolini, Enrico Dall'Ara

**Affiliations:** ^1^Department of Oncology and Metabolism, Mellanby Centre for Bone Research, University of Sheffield, Sheffield, United Kingdom; ^2^INSIGNEO Institute for in silico Medicine, University of Sheffield, Sheffield, United Kingdom; ^3^Department of Industrial Engineering, Alma Mater Studiorum - Università di Bologna, Bologna, Italy

**Keywords:** nanoindentation, bone, mouse tibia, indentation properties, reduced modulus, hardness

## Abstract

The local spatial heterogeneity of the material properties of the cortical and trabecular bone extracted from the mouse tibia is not well-known. Nevertheless, its characterization is fundamental to be able to study comprehensively the effect of interventions and to generate computational models to predict the bone strength preclinically. The goal of this study was to evaluate the nanoindentation properties of bone tissue extracted from two different mouse strains across the tibia length and in different sectors. Left tibiae were collected from four female mice, two C57BL/6, and two Balb/C mice. Nanoindentations with maximum 6 mN load were performed on different microstructures, regions along the axis of the tibiae, and sectors (379 in total). Reduced modulus (*Er*) and hardness (*H*) were computed for each indentation. Trabecular bone of Balb/C mice was 21% stiffer than that of C57BL/6 mice (20.8 ± 4.1 GPa vs. 16.5 ± 7.1 GPa). Moreover, the proximal regions of the bones were 13–36% less stiff than the mid-shaft and distal regions of the same bones. No significant differences were found for the different sectors for *E*_*r*_ and *H* for Balb/C mice. The bone in the medial sector was found to be 8–14% harder and stiffer than the bone in the anterior or posterior sectors for C57BL/6 mice. In conclusion, this study showed that the nanoindentation properties of the mouse tibia are heterogeneous across the tibia length and the trabecular bone properties are different between Balb/C and C57BL/6 mice. These results will help the research community to identify regions where to characterize the mechanical properties of the bone during preclinical optimisation of treatments for skeletal diseases.

## Introduction

Bone diseases such as osteoporosis affect the quality of life of millions of patients every year worldwide. Preclinical assessment of bone anabolic or anti-resorptive interventions in mice models is fundamental to understand their efficacy before clinical trials (Bouxsein et al., 2010).

A combination of *in vivo* micro computed tomography imaging and finite element models can be used to evaluate morphometric, densitometric, and mechanical properties of the mouse tibia (Razi et al., 2015; Lu et al., 2017). The optimisation of longitudinal imaging and computational methods to evaluate non-invasively the mechanical properties of bone has the potential to dramatically reduce the number of rodents to be used in preclinical musculoskeletal research, in line with the 3Rs (replacement, refinement, and reduction of animals in research; Viceconti and Dall'Ara, 2019). Nevertheless, little is known about the local mechanical properties of the tissue in different mice of different mouse strains. Moreover, the material properties in the finite element models are assigned by using local tissue properties (i.e., the Elastic Modulus) measured in back-calculated studies for the caudal vertebra of one strain of mice (Webster et al., 2008; Oliviero et al., 2018). Alternatively, such properties could be assigned with local nanoindentation measurements (Wolfram et al., 2010).

Nanoindentation can be used to measure the local tissue moduli and hardness of biomaterials (Zysset et al., 1999; Zysset, 2009). This technique has been used intensively to study the mechanical properties of bovine (Lucchini et al., 2011; Carnelli et al., 2013; Dall'Ara et al., 2015) and human (Zysset et al., 1999; Spiesz et al., 2013) bone tissue. Moreover, some studies have characterized the local mechanical properties of the mouse bone tissue, which is important to study the effect of bone diseases and related interventions in preclinical studies. For example, the nanoindentation properties of mouse bone tissue have been evaluated in the healing callus after fracture of the femur (Leong and Morgan, 2008, 2009). Furthermore, nanoindentation tests have been performed in the femoral cortical bone of 22 weeks old female (Casanova et al., 2017) C57BL/6 mice or 16 weeks old (gender not reported) C57BL/6 (Pathak et al., 2011; with dynamics nanoindentation) and A/J (Pathak et al., 2012) mice (with spherical indenter).

The evaluation of tibia properties is very important as it is a typical anatomical site to access the effect of musculoskeletal interventions in murine studies (Bouxsein et al., 2010) and this peripheral site is usually used for *in vivo* assessment of bone remodeling with *in vivo* micro computed tomography (Dall'Ara et al., 2016). However, only a few studies have measured the nanoindentation properties of the cortical bone in the tibia diaphysis: in 9 weeks old female C57BL/6 mice (Rodriguez-Florez et al., 2013); in 4 months old female C57BL/6J, DBA/2J, C3H/HeJ mice (Akhter et al., 2004); in 7 weeks old male B6C3Fe-a/aCol1a2oim/oim, Phospho1-R74X null mutant and respective wild type controls (Rodriguez-Florez et al., 2015); in 10 weeks old male C57BL/6J mice after nephrectomy or sham operation (Heveran et al., 2016); in 4 and 12 months old SAMP6 and SAMR1 mice (Silva et al., 2004); and in C3H and B6C3H-F_2_ mice of different ages (Jiao et al., 2007). Nanoindentation measurements revealed that the elastic modulus is lowest in DBA/2J mice (22.9 GPa) compared to C3H/HeJ and C57BL/6J that showed similar values (28.3 and 30.9 GPa) (Akhter et al., 2004); that both fragile (B6C3Fe-a/aCol1a2oim/oim null mutant) and ductile (Phospho1-R74X null mutant) disease models lead to a 19% and 14% reduction in elastic modulus compared to control, respectively (Rodriguez-Florez et al., 2015); that local material properties of the cortical bone were impaired for mice after nephrectomy (Heveran et al., 2016); that a senescence accelerated mouse strain (SAMP6) has stiffer and harder cortical bone compared to SAMR1 controls (Silva et al., 2004); and that the bone of C3H mice reaches peaks in elastic modulus and hardness at 4 months of age (Jiao et al., 2007). While the bone material properties from different mouse inbreed strains have been tested, only one study in the literature has evaluated the indentation properties of Balb/C mice bone tissue, showing that the bone tissue in adolescent mice increased in function of age until 40 days of age for which similar elastic modulus was found compared to adult mice (450 days old; Miller et al., 2007). The fact that only one study has measured the nanoindentation properties of bone from Balb/C mice is surprising considering that these mice, together with C57BL/6 mice, are the most common animal models for studying the effect of interventions on skeletal health. These mice strains were shown to have different morphological properties and remodeling patterns after ovariectomy (Roberts et al., 2019), and different response to passive mechanical loading (Holguin et al., 2013) or bone interventions (Lynch et al., 2011). Nevertheless, it is still unknown if the indentation properties of the mouse tibia of these two strains are different. Moreover, it is not known how heterogeneous the nanoindentation properties across the tibia of the mouse are. This property of bone is fundamental to better understanding the link between bone morphometric, densitometric, and mechanical properties and therefore evaluating the effect of skeletal diseases and related interventions in preclinical studies.

The aim of this study was to evaluate the mechanical properties using nanoindentation in different regions of the mouse tibia extracted from skeletally mature C57BL/6 or Balb/C mice.

## Materials and Methods

### Sample Preparation

Four left mouse tibiae (two from C57BL/6 and two from Balb/C mice, 16 weeks old, female) were collected from a previous study in which the mice were euthanized with cervical dislocation (Roberts et al., 2019). The tissues used in this study were collected from previous animal work, performed under a British Home Office project license (PPL 40/3499) and in compliance with the UK Animals (Scientific Procedures) Act 1986. The whole legs were stored fresh frozen (−20°C) after the animals were culled. At the beginning of this study, the left legs were thawed at room temperature, the tibiae were dissected and then stored in freezer until further processing (−20°C). The tibiae were defrosted in 0.9%NaCl saline solution. They were left 2 h in air at room temperature for dehydration, and then they were embedded in acrylic resin (EpoFix, Struers, Catcliffe, UK). A vacuum procedure was used to reduce the amount of bubbles in the external surface of the bone but at the same time avoiding the infiltration of the resin within the nano-porosities such as osteocytes lacunae and canaliculi (Dall'Ara et al., 2013).

**Figure 1:**
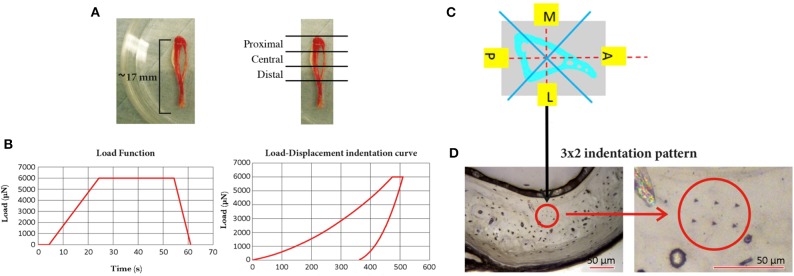
Mouse tibia and the three sections in which it was sectioned (Proximal, Central, and Distal); **(B)** the loading procedure used for the nanoindentation tests (left, maximum load of 6,000 μN, loading time of 20.00 s, unloading time of 6.65 s, and holding time of 30.00 s) and a typical Load-Displacement indentation curve (right); **(C)** schematic representation of the four sectors, in one slice of the tibia, where the indentations were performed; **(D)** typical 3 × 2 indentation pattern on the lateral section of the cortical bone of one slice of the tibia (left) and a magnification of the pattern (right).

From each tibia three 3 mm thick sections perpendicular to the tibia longitudinal axis were isolated (Proximal, Central, and Distal) by using a low speed diamond saw (IsoMet, Buehler, Germany). Each specimen was polished by using three silicon carbide papers with decreasing grain size (P400, P800, and P1200, Struers, Willich, Germany) followed by a polishing step with alumina particles (0.05 μm, MasterPrep, Buehler, Germany), Figure 1.

### Nanoindentation Tests

For each section 24 indentations were performed on the cortical bone, divided in four groups in the medial, lateral, anterior, and posterior regions of the tibia (six indentations per region in the central part of the cortical bone, avoiding the circular lamellae close to the endosteum, and periosteum). Considering that in the mouse tibia trabeculae are located only in the proximal portion of the bone, for each specimen five trabeculae distributed across the whole most proximal region were chosen and three to five indentations per trabecula were performed according to its length. The distance between the indentations within the same matrix (2 × 3 for the cortical bone, 1 × 3–5 for trabecular bone) was 15 μm and the distance from the edge was at least 30 μm.

Indentations were performed with a Berkovich tip (Hysitron TI Primer nanoindenter, Bruker, USA) up to a maximum load of 6,000 μN that on our specimens lead to a penetration depth of ~500 nm and radius of ~3,500 nm (indentations within a lamella), as reported in a previous study where indentations were performed on the mouse femur (Casanova et al., 2017). The indentations were performed with the following parameters: loading time equal to 20.0 s (loading rate equal to 300 μN/s), holding time equal to 30.0 s, and unloading of 6.65 s (unloading rate equal to 902 μN/s). In total, 386 indentations were performed (288 on the cortical bone and 98 on the trabecular bone). Each load-displacement indentation curve and each image of the indentation was checked for potential contact issues or other problems.

For each indentation the reduced modulus (*E*_*r*_), the indentation modulus (*E*_*ind*_), the elastic modulus assuming a Poisson's ratio of 0.3 (Zysset, 2009) for the bone (*E*_*b*_) and the Hardness (*H*) were computed by using the Oliver and Pharr method (Oliver and Pharr, 1992):

(1) $$\begin{array}{l} E_{r}=\frac{\sqrt{\pi }}{2\beta \sqrt{A_{c}}}\frac{dP}{dh}\left(h_{\max}\right) \end{array}$$

where β is an empirical indenter shape factor, *A*_*c*_ is the indentation projected area and *dP/dh* is the slope of the load-displacement curve at the maximum depth (*h*_*max*_).

(2) $$\begin{array}{l} E_{ind}=\;\frac{E_{r}E_{t}}{E_{t}-\;E_{r}\left(1-\nu_{t}^{2}\right)} \end{array}$$

where *E*_*t*_ and ν_*t*_ are the elastic modulus and the Poisson ratio of the tip, respectively and *E*_*r*_ is the reduced modulus.

(3) $$\begin{array}{l} E_{b}=\;\frac{\left(1-\nu_{b}^{2}\right)\;E_{r}E_{t}}{E_{t}-E_{r}\;\left(1-\nu_{t}^{2}\right)} \end{array}$$

where *E*_*r*_ and *E*_*t*_ are the reduced modulus and the indentation modulus and ν_*t*_and ν_*b*_ are the Poisson ratios of the tip and of the bone sample, respectively.

(4) $$\begin{array}{l} H=\frac{P_{\max}}{A} \end{array}$$

where *P*_*max*_ is the peak indentation load and *A* is the projected area of the impression.

The three moduli (*E*_*r*_, *E*_*ind*_, and *E*_*b*_) have been reported in order to simplify the comparison between the results obtained in this study and those reported in the literature.

### Statistics

The significance of the effect of different factors on *E*_*r*_ and *H* was tested (IBM SPSS software, SPSS Statics Version 25). The parameters were not normally distributed (Kolmogorov-Smirnov, *p* < 0.05). Therefore, the non-parametric Kruskal-Wallis tests were used (significance threshold equal to 0.05). If a factor was significant, a Bonferroni *post-hoc* analysis was performed.

For cortical bone properties significance was investigated for the following factors: mouse Strains (Balb/C vs. C57BL/6), Regions (Distal, Central, and Proximal), and Sectors (Anterior, Medial, Posterior, and Lateral). For the trabecular bone, the effect of mouse Strains (Balb/C vs. C57BL/6) was analyzed. Differences between proximal cortical bone and proximal trabecular bone were analyzed.

## Results

Of the 386 indentations curves seven were excluded due to contact problems. Therefore, a total of 379 indentations were analyzed (284 on cortical bone and 95 on trabecular bone). Results and statistics are reported here only for *H* and *E*_*r*_. The results for *E*_*ind*_ and *E*_*b*_ are reported in the Supplementary Material. Nanoindentation data are available within the figshare repository: https://doi.org/10.15131/shef.data.11309678.v2.

### Effect of “Mouse Strain”

For cortical bone, small but significant differences in *E*_*r*_(*p* = 0.032) and no significant differences in *H* (*p* = 0.812) were observed between the two mouse strains. The trabecular bone of Balb/C mice was stiffer (+20.6%, *p* < 0.001) and harder (+27.1%, *p* < 0.001) than that of C57BL/6 mice (Table 1).

**Table 1:** Results from the indentations performed on the cortical and trabecular bone for the two mouse strains.

**Effect of “mouse strain”**				
**Mechanical properties**	**Bone Type**	**C57BL/6**	**Balb/C**	**Difference**
*E_*r*_* (GPa)	Cortical	25.04 ± 6.18	24.29 ± 5.24	−3.0%^*^
*H* (GPa)	Cortical	0.90 ± 0.21	0.93 ± 0.19	+3.2% NS
*E_*r*_* (GPa)	Trabecular	16.50 ± 7.10	20.79 ± 4.12	+20.6%^**^
*H* (GPa)	Trabecular	0.62 ± 0.27	0.85 ± 0.18	+27.1%^**^

* indicates p < 0.05;

** indicates p < 0.001; “NS” indicates p > 0.05.

*Data is reported as mean ± standard deviation including both tibiae for each strain and all sections*.

### Effect of “Region”

**Figure 2:**
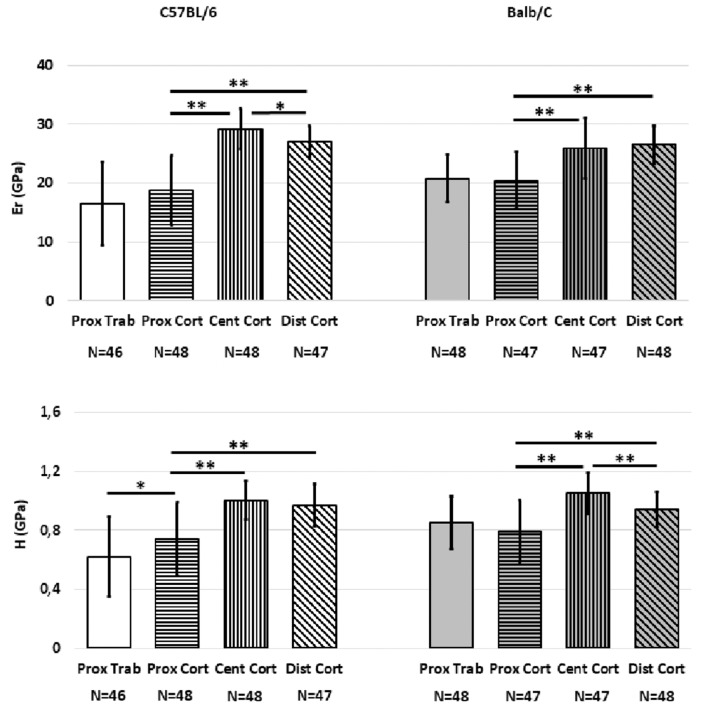
Mean values of *E*_*r*_ and *H* from the indentations performed on the cortical and trabecular bone for the two mouse strains split for the different regions (Proximal, Central, Distal). Error bars represent standard deviation. *indicates *p* < 0.05; **indicates *p* < 0.001.

Significant differences were associated with “Region” for most mechanical properties (Figure 2). For C57BL/6 strain *E*_*r*_ was higher in the central (36.0%, *p* < 0.001) or distal (30.7%, *p* < 0.001) regions compared to the proximal region. Significant differences were found between the *E*_*r*_ of the central and distal regions (7.6%, *p* = 0.001). Similar trends were found for *H* (26% difference between proximal and central regions and 23.7% difference between proximal and distal regions, *p* < 0.001 for both); no difference between the central and distal regions was found (*p* = 0.393). The *H* in the proximal cortical bone was higher than that in the proximal trabecular bone (16.2% difference, *p* = 0.014). However, *E*_*r*_ values in the proximal trabecular and cortical bone were similar (*p* = 0.111).

For Balb/C mice, the bone tissue in the central and distal regions was found to be stiffer (+21.1 and +22.9%, respectively) and harder (+24.8 and +16%, respectively) than that in the proximal region (*p* < 0.001 in all cases). Similar values of *E*_*r*_ (*p* = 0.224) but higher values of *H* (+10.5%, *p* = 0.003) were found in the bone of the central region compared to that in the distal region. Moreover, no differences were found in *E*_*r*_ or *H* between the proximal cortical bone and the proximal trabecular bone (*p* > 0.369).

### Effect of “Sector”

**Figure 3:**
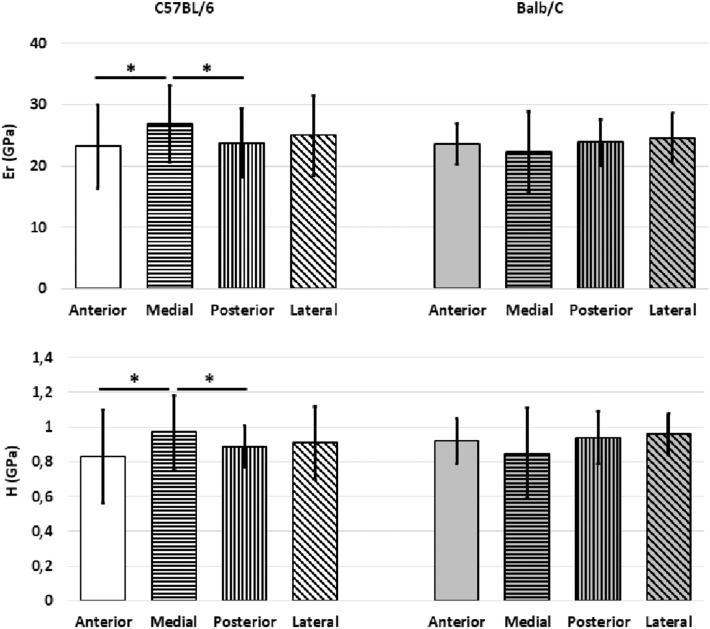
Mean values of *E*_*r*_ and *H* from the indentations performed on the cortical bone for the two mouse strains split for the different sectors (Anterior, Medial, Posterior, Lateral). Error bars represent standard deviation. *indicates *p* < 0.05.

In C57BL/6 mice the bone tissue in the medial sector was found to be stiffer and harder than that in the posterior (+11.1 for *Er, p* = 0.005; +8.2% for *H, p* = 0.006) and anterior (+13.2% for *Er, p* = 0.006; +14.4% for *H, p* = 0.006) sectors (Figure 3).

In Balb/C mice similar stiffness and hardness values were found for the bone of the different sectors (*p* = 0.566 for *E*_*r*_ and *p* = 0.427 for *H*) (Figure 3).

## Discussion

The goal of this study was to characterize the heterogeneous regional bone material properties along the tibia in different sectors, for C57BL/6 and Balb/C mice, by using nanoindentation.

The trabecular bone was found to be significantly stiffer and harder in the Balb/C mice compared to tissue extracted from C57BL/6 mice. Conversely, the cortical bone was stiffer in C57BL/6 mice, but similar values of *H* were found for the two strains. The difference in stiffness between the bone tissue extracted from the two strains is probably due to differences in local mineralization, which drives the elastic response of the tissue (Bala et al., 2011). Considering that the bone tissue was extracted from skeletally mature but young mice (16 weeks), the intrinsic heterogeneity of the mineralization of the bone structural units may play a role, especially in the most metabolically active trabecular bone. These differences should be considered when generating finite element models, fundamental for the reduction, and partial replacement of the use of mice in musculoskeletal research (Viceconti and Dall'Ara, 2019). Furthermore, significant differences were found between different regions of the tibia with the proximal cortical bone being less stiff than the central and distal regions for both strains. This result may be explained by the localization of younger and less mineralised tissue in the region closer to the growth plate (Buie et al., 2008; Willie et al., 2013). For C57BL/6 the cortical bone in the proximal region was found to be harder and stiffer (only a trend) than the trabecular bone in the proximal portion of the tibia. This is in line with previous results obtained on human tissue (Zysset et al., 1999). Conversely, for Balb/C no differences were found between mechanical properties of the proximal cortical and trabecular bone tissue. This difference may be due to the different growth of the mice and the local mineralization of the cortical and trabecular bone tissue.

Similar values of mechanical properties were found across the different sectors, with only significant differences for C57BL/6 mice between medial and posterior or anterior bone tissue. This difference may be due to the asymmetry in bone remodeling activities as observed with *in vivo* microCT imaging of the mouse tibia (Roberts et al., 2019). However, it needs to be further explored if this difference can be associated with the loading condition due to the effect of the curvature of the bone, as under physiological compressive loads a peak of compressive strains is located on the medial surface of the tibia below the tibio-fibular junction (Oliviero et al., 2018; Cheong et al., 2020). Nevertheless, this difference was not observed in Balb/C mice, which have a similar geometry of the mouse tibia but lower curvature. Interestingly a previous study performed on 24–25 weeks old mice that underwent voluntary exercise showed that the bone of the posterior and anterior sectors of the femur was stiffer (higher Er) than that in the medial and lateral sectors (Middleton et al., 2010). The differences between the results of these studies could be due to the different studied anatomical sites and to the different age of the animals.

Similar values of elastic modulus and hardness were reported for nanoindentations on C57BL/6 mouse tibia (Rodriguez-Florez et al., 2013): the differences in *E*_*r*_ and *H* between the two studies were ~30% (C57BL/6 in the central region of the cortical bone), and could be explained by differences in the age of the animals (9 weeks old), methods of dehydration (in ethanol series vs. at room temperature), the indentation parameters (max load 8 mN), and the indentation position (only midshaft). Another study carried out on the cortical bone of C57BL/6 mice (Akhter et al., 2004) showed a difference of 17% in *E*_*b*_ and of 23% in *H* compared to this study, that may be due to different indentation parameters (faster loading/unloading rates).

In this study a similar nanoindentation procedure was used as reported by Casanova et al. (2017) for indentations on the mouse femur; while the indentation parameters were the same, the anatomical site, the age and the condition of the tissue were different. As expected, there was a difference of ~26% between the *E*_*r*_ found by Casanova et al. (mouse femur, C57BL/6, female 22 weeks old, hydrated) and that found in this study (mouse tibia, C57BL/6, female 16 weeks old, dehydrated). Considering that previous studies reported that the reduced modulus and hardness are 20–30% lower in the rehydrated specimens (Wolfram et al., 2010), the differences in indentation properties may be mainly due to the different tissue conditions. Nevertheless, the difference in age and anatomical site may also play a role in the mineralization of the tissue and, therefore, in its local stiffness.

The main limitation of this study is the small sample size, which should be increased in the future in order to generalize the findings for different ages and gender. Nevertheless, the small sample size allowed for a detailed characterization of the trabecular bone and of the cortical bone in different regions and sectors. Moreover, bone has been considered as locally isotropic and indentations were performed only along the bone axial direction. It would be interesting in the future to extend these analyses on properties along the circumferential and radial directions (Dall'Ara et al., 2013).

## Conclusion

In conclusion, the results of this study have highlighted that there are differences in local material properties measured with nanoindentation between the proximal and the other regions of the mouse tibia extracted from C57BL/6 and Balb/C female mice. These results should be considered when generating computational models of the mouse tibia and when evaluating the effect of novel interventions on the local material properties of the tibia, which should be analyzed in matched positions.

## Data Availability Statement

The data related to this study can be accessed fully here: https://doi.org/10.15131/shef.data.11309678.

## Author Contributions

SO, LC, and ED'A: data acquisition. VP: analysis and interpretation of the data. VP, SO, LC, and ED'A: drafting and/or revision of the article. All authors have reviewed and agreed upon the last version of the manuscript and involved in the conception and design of the study.

## Conflict of Interest

The authors declare that the research was conducted in the absence of any commercial or financial relationships that could be construed as a potential conflict of interest.
