# Supplementary material for: Regional Nanoindentation Properties in Different Locations on the Mouse Tibia From C57BL/6 and Balb/C Female Mice
Source: Front Bioeng Biotechnol. 2020 May 15;8:478. doi: 10.3389/fbioe.2020.00478 (PMC7243342; doi:10.3389/fbioe.2020.00478)
Supplement: Supplementary file 1 [file Table_1.DOCX]

## Supplementary Figures

##
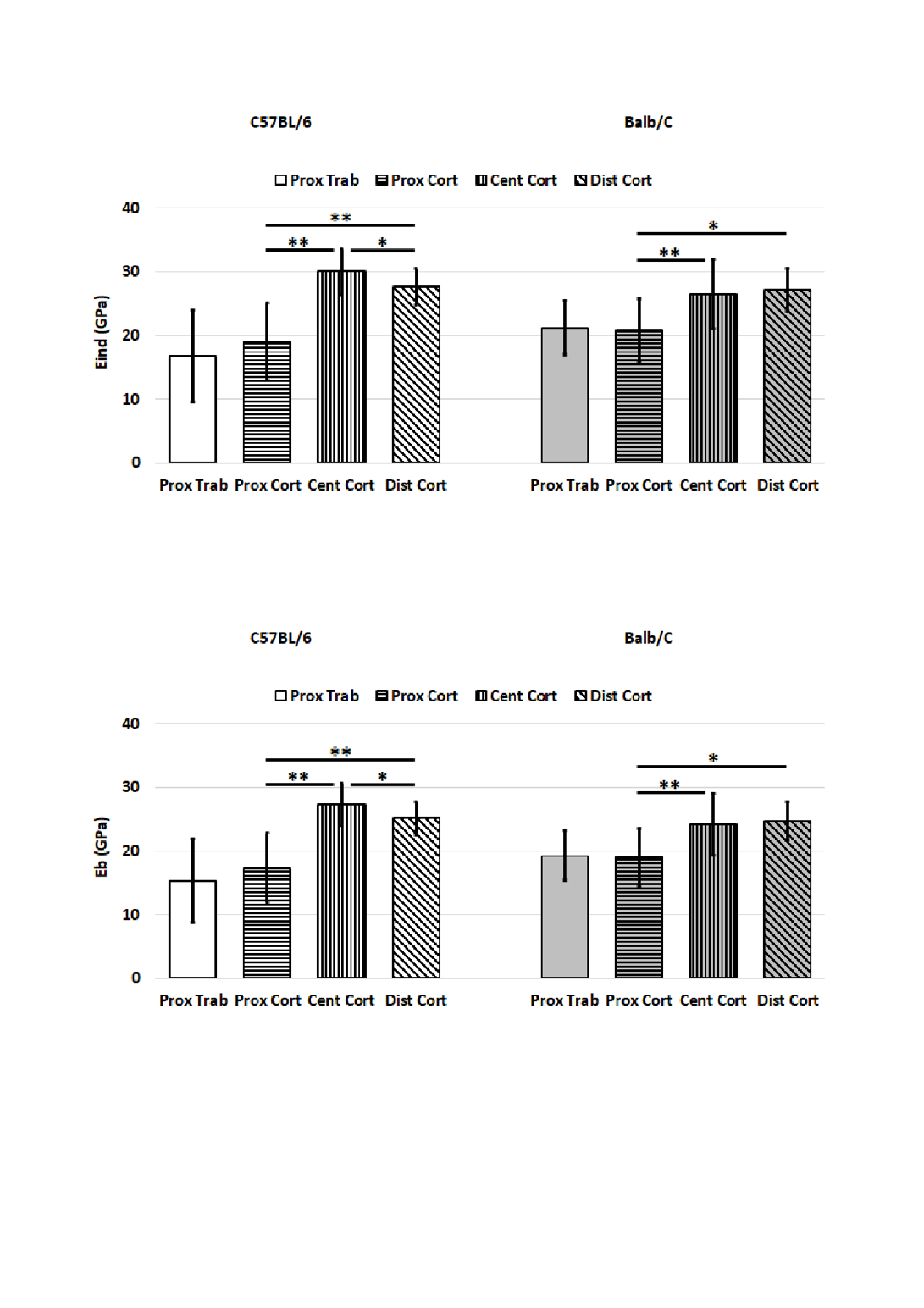


## Supplementary Figure 1. Mean values of Eind and Eb from the indentations performed on the cortical and trabecular bone for the two mouse strains split for the different regions (Proximal, Central, Distal). Error bars represent standard deviation. * indicates p-value<0.05; ** indicates p-value<0.001

**
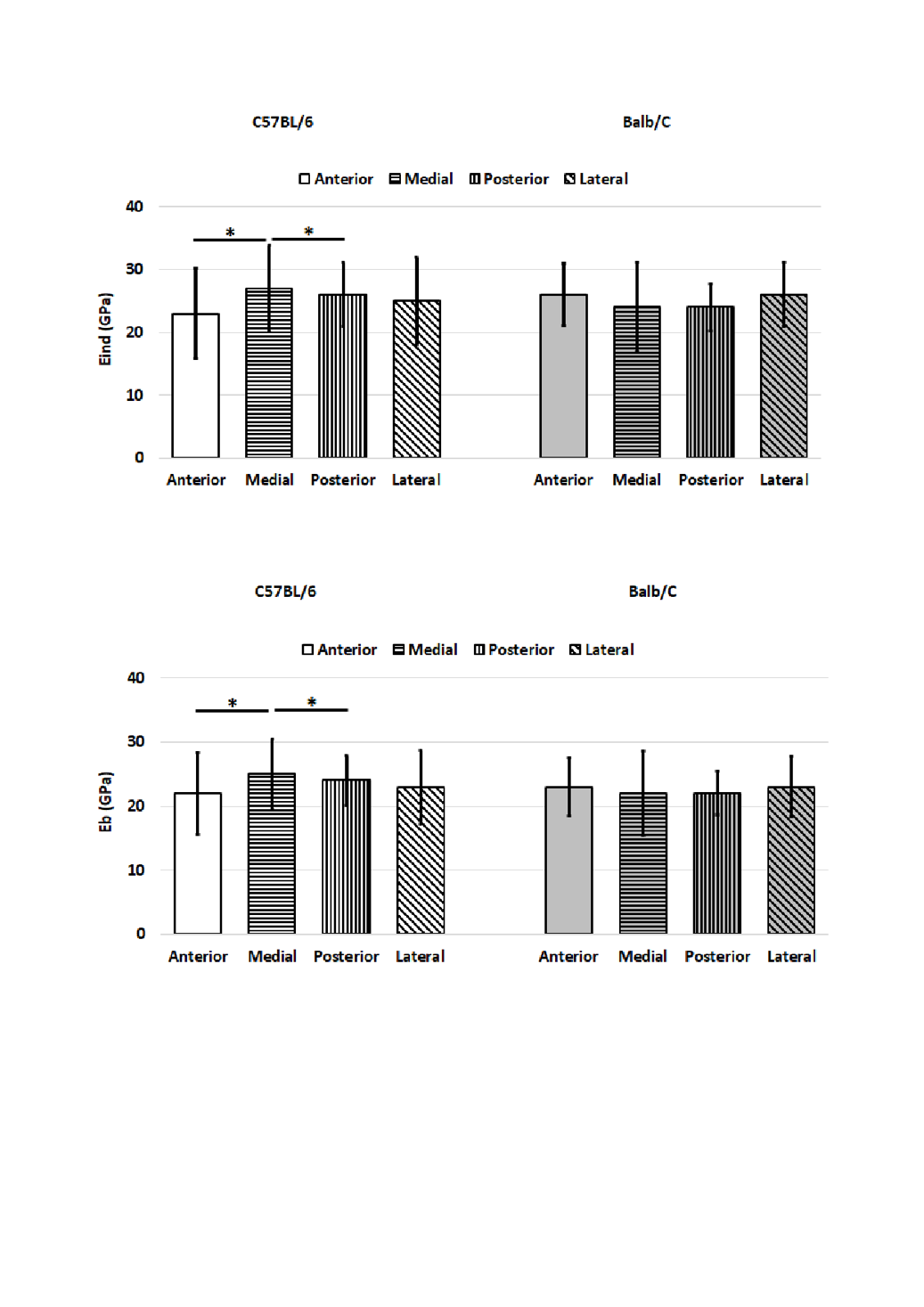
**

**Supplementary Figure 2.** Mean values of Eind and Eb from the indentations performed on the cortical bone for the two mouse strains split for the different sectors (Anterior, Medial, Posterior, Lateral). Error bars represent standard deviation. * indicates p-value<0.05
